# Escape room design in training crew resource management in acute care: a scoping review

**DOI:** 10.1186/s12909-024-05753-z

**Published:** 2024-07-30

**Authors:** Gerald J. Jaspers, Simone Borsci, Johannes G. van der Hoeven, Wietske Kuijer-Siebelink, Joris Lemson

**Affiliations:** 1 Human Factors and Cognitive Ergonomics, Research Coordinator Human Factors-CODE team DSI institute & Faculty Behavioral, Management and Social Sciences (BMS), Data and Education (CODE) Group, University of Twente, Drienerlolaan 5, Cognition, Enschede, NB 7522 The Netherlands; 2 Department of Surgery and Cancer, NIHR London IVD, Imperial College of London, London, UK; 3 Department of Intensive Care, Radboud University Medical Center, Geert Groote Plein Zuid 10, Nijmegen, 6525GA The Netherlands; 4 Research on Learning and Education, Radboudumc Health Academy, Radboud University Medical Center, Geert Groote Plein Zuid 10, Nijmegen, 6525GA The Netherlands; 5 School of Education, HAN University of Applied Sciences, Nijmegen, The Netherlands

**Keywords:** Crisis resource management, Teamwork, Non-technical skills, Escape game, Puzzle room

## Abstract

**Background:**

Effective teamwork is crucial to providing safe and high-quality patient care, especially in acute care. Crew Resource Management (CRM) principles are often used for training teamwork in these situations, with escape rooms forming a promising new tool. However, little is known about escape room design characteristics and their effect on learning outcomes. We investigated the current status of design characteristics and their effect on learning outcomes for escape room-based CRM/teamwork training for acute care professionals. We also aimed to identify gaps in literature to guide further research.

**Methods:**

Multiple databases were searched for studies describing the design and effect of escape rooms aimed training CRM/teamwork in acute care professionals and in situations that share characteristics. A standardized process was used for screening and selection. An evidence table that included study characteristics, design characteristics and effect of the escape room on learning outcomes was used to extract data. Learning outcomes were graded according to IPE expanded typology of Kirkpatrick’s levels of learning outcome and Medical Education Research Study Quality Instrument (MERSQI) scores were calculated to assess methodology.

**Results:**

Fourteen studies were included. Common design characteristics were a team size of 4–6 participants, a 40-minute time limit, linear puzzle organization and use of briefing and structured debriefing. Information on alignment was only available in five studies and reporting on several other educational and escape room design characteristics was low. Twelve studies evaluated the effect of the escape room on teamwork: nine evaluated reaction (Kirkpatrick level 1; *n* = 9), two evaluated learning (Kirkpatrick level 2) and one evaluated both. Overall effect on teamwork was overtly positive, with little difference between studies. Together with a mean MERSQI score of 7.0, this precluded connecting specific design characteristics to the effect on learning outcomes.

**Conclusions:**

There is insufficient evidence if and how design characteristics affect learning outcomes in escape rooms aimed at training CRM/teamwork in acute care professionals. Alignment of teamwork with learning goals is insufficiently reported. More complete reporting of escape rooms aimed at training CRM/teamwork in acute care professionals is needed, with a research focus on maximizing learning potential through design.

**Supplementary Information:**

The online version contains supplementary material available at 10.1186/s12909-024-05753-z.

## Background

Effective teamwork is crucial to providing safe and high-quality patient care, especially in acute care settings where stakes are high and time-sensitive decisions and actions are required. In the past two decades, there has been a growing interest in training teamwork in these settings and its effectiveness [1, 2]. Crew (or Crisis) Resource Management (CRM) principles are frequently used for structuring, improving and training teamwork and communication in these settings [1]. CRM identifies factors in, and threats to effective teamwork and offers tools to improve teamwork and communication and prevent error. Substantial evidence shows that training improves CRM skills in health care professionals on multiple learning outcome levels and might lead to safer care [3]. To achieve these outcomes effective training is necessary [3–7]. CRM training varies considerably and can include a wide range of interventions, like lectures, table-top games, simulation, etc. [4]. Practice-based interventions, like simulation, are often included in CRM training. CRM skills are trained by applying in simulation and this was found to be more effective than other instructional methods [8–10].

Other practice-based interventions might also be able to fulfill this role. A new, innovative and practice-based training tool in CRM/teamwork training is the use of escape rooms. Escape rooms are, as defined by Nicholson [11] ‘live-action team-based games where players discover clues, solve puzzles, and accomplish tasks in one or more rooms in order to accomplish a specific goal (usually escaping from the room) in a limited amount of time’. They offer great potential for teamwork training in acute care as they are, by definition, time-limited team-based activities that both force and facilitate teamwork, with the need to coordinate tasks and communicate [11]. Their use in healthcare, as well as in education in general, has increased significantly in recent years [12, 13]. The high learning potential is also reflected in that they are often enjoyed by participants and, albeit limited, in healthcare students have shown an increase in skills, knowledge, and attitudes [12].

Educational intervention design characteristics may affect learning outcomes. Several considerations have been proposed for the design requirement for developing educational escape rooms in healthcare settings in general [14, 15] and also for teamwork [16], but such considerations are often based on theory and practice experience, and not on a synthesis of the available evidence. There seems to be consensus that alignment of the learning goals with the escape room is an important requirement. Recent reviews in escape rooms in healthcare students [12] and higher education (including medical escape rooms) [17] underscores this, but also identify a lack of evidence on the impact design characteristics have on learning outcomes [12]. Little to no guidelines are available regarding how escape rooms that aim at improving CRM/teamwork for the acute care setting should be designed, or how to maximize learning outcomes through design in this setting. Therefore, the question driving our review was: what are the design requirements that should be taken into account in the design of such escape rooms.

In the present study, we aim to identify common design characteristics, relate these to learning outcomes and thereby identifying a set of evidence-based design requirements for escape rooms to train CRM/teamwork in acute care. To achieve these goals, we performed a scoping review of the literature regarding design characteristics and their effect on learning outcomes in escape rooms used for crew resource management and/or teamwork training for healthcare professionals in acute care settings.

## Methods

### Research questions

To guide the development of physical escape rooms aimed at improving crew resource management/teamwork in healthcare personnel in acute care settings, the present review investigated the answer to the following questions:

1. Which common design characteristics can be derived from peer-reviewed literature?
2. What common design characteristics can be derived from similar situations:
  Escape rooms assessing students instead of healthcare personnel.
  Virtual escape rooms instead of physical escape rooms.
  Escape rooms used for training CRM in situations with similar characteristics to acute care: time-limited, high stakes, high-pressure, high safety (i.e., aviation, military, etc.)
3. When connecting design characteristics to learning outcomes, which design requirements can be identified that maximize learning outcomes?
4. What are the major gaps in the evidence on design requirements for optimizing learning outcomes?

To best meet these broad objectives, and analyze a range of different study designs, we used a scoping review design. The review was conducted following the Preferred Reporting Items for Systematic Reviews and Meta-analysis Protocols extension for scoping reviews (PRISMA-ScR) [18, 19].

### Eligibility criteria

Inclusion criteria were drafted to match the primary aim: *studies describing the design of physical escape rooms* to *train crew resource management* in *healthcare professionals* working in *acute care*. As CRM is about teamwork, all studies on escape rooms aimed at teamwork, or related terms were included. Escape rooms can have other learning goals (i.e., knowledge or skills) besides teamwork that influences design. To be included, training CRM/teamwork had to be one of the main aims. If studies had a different focus than describing design, they were included if sufficient detail on design was provided. This was defined as at least a puzzle scheme and description of the puzzle organization.

To enrich the data, we also looked at studies describing escape rooms in situations that share characteristics. To balance between precision on the one hand, and not miss relevant publications on the other, studies that differed on one aspect of the primary aim were included. This was defined as studies describing acute care escape rooms, but only fulfilling 2 of the 3 other criteria: (i) virtual instead of a physical escape room; (ii) students instead of healthcare professionals; (iii) Settings with similar characteristics to acute care: time-limited, high stakes, high-pressure, high safety (for example aviation, military). These studies were grouped as ‘virtual’, ‘students’, and ‘setting’ and are mentioned throughout the review as such.

Only studies that were full-text, empirical and published in a peer-reviewed journal were included. There was no limitation on study type or design (i.e. qualitative, qualitative) but we excluded letters to the editor, conference papers/abstract, etc. because of insufficient detail. Finally, we included studies that did not describe design, but measured effectiveness/evaluation of an escape room of which the design was described in an included article. These studies were used for the analysis of the effect of the design criteria on learning outcomes.

### Databases and search strategy

For full information on the selection of databases and the search strategy, we refer to additional file 1. An extensive range of databases were searched (CINAHL, EMBASE, ERIC, MEDLINE (PubMed), PsycINFO, Scopus, and Web of Science). To cross-check no relevant articles were missed, three additional sources were used: (1) Forward and backward citation tracking of articles eligible for inclusion, (2) Elicit [20] (an AI tool that uses language models to find relevant papers), (3) Google Scholar [21].

The search strategy was drafted by one author (GJ) and iteratively refined through team discussion. A librarian, experienced in systematic searches, evaluated and further refined the search strategy.

The search focused on two keywords: escape room and Crew Resource Management/teamwork. Both keywords were maximally broadened. For escape room, we added all alternative and related terms. For crew resource management/teamwork, we also included related and alternative terms. Additionally, we added CRM-elements like situational awareness, communication, leadership, task allocation, and decision-making. Databases were checked for relevant MeSH (or equivalent) terms and additionally, all terms were used in a title, abstract, and keyword search. No language restrictions were applied, but the search was limited to studies published after the year 2000, as the first well-documented escape room was not described until 2007 [11]. The search was completed in June 2023. Results were imported into EndNote (EndNote™, version 20, Clavirate, Philadelphia, U.S.). After removing duplicates the results were exported to Rayyan [22] for screening.

Title and abstract of all articles were independently screened for eligibility by two authors (GJ and JL). Any discrepancies were solved through discussion. In the second stage, two authors (GJ and JL) screened the full texts of all included articles against the eligibility criteria; any discrepancies were resolved in discussion with a third author (SB).

### Data items and charting process

Using an iterative process, a set of data items to extract was defined. First three categories were determined in which to categorize and structure the data and reporting:

- Study characteristics.
- Educational and escape room design characteristics.
- Effect on learning.

Specific data extraction items in these categories were identified through studying reviews and frameworks: educational escape room design [14–17, 23–26], healthcare CRM training/simulation [4, 27, 28], and interprofessional education [29]. All authors critically reviewed the data-items, added or deleted items and through iterative discussion, a final selection was made. In Additional file 2 the full list of data-items can be found as column headings.

In ‘study characteristics’, data were collected on subjects, setting and aim of the study and escape room and was used to provide an overview of the included studies. In the ‘educational and escape room design characteristics’, data that guided the design were extracted. Alignment of learning goals with the escape room is considered essential in design [16, 17]. We used this data item to extract data on explicit information how alignment was achieved. However, all design characteristics relate to alignment. These separate characteristics were identified using the process mentioned above. For example, for the educational underpinning items like learning theory and CRM/teamwork model, briefing and debriefing technique were extracted. As teams in acute care often consist of members with different roles and from different backgrounds, the interprofessional educational (IPE) characteristics [29] interdependence (the need for a contribution from the expertise of all team-members [29]) and embodiment (also called immersion; the feeling of being immersed in a situation that feels authentic and that is similar to working in their profession) were included. Also data on the escape room design characteristics were extracted, such as team size, puzzle organization (open, linear/sequential, path-based, pyramid or complex (for more info and graphical representation see reference [11]) and facilitator role.

In the ‘effect on learning’ category, data were collected on the effect of the escape room on teamwork. To define design requirements, this data was used link the design characteristics to their effect on learning outcomes. As our focus is on CRM/teamwork, only data on the effect of the escape room on teamwork were extracted and data on, for example, knowledge or skills was not extracted. The level of the evaluation on the effect on CRM/teamwork was determined using Reeves’ [30] IPE expanded typology of Kirkpatrick’s [31] classic model of the levels of learning outcomes: reaction (level 1); modification of attitudes/perceptions (level 2a); acquisition of knowledge/skills (level 2b); behavioral change (level 3); outcome on a patient or organizational level (level 4).

To appraise the methodological quality of the teamwork evaluation, Medical Education Research Study Quality Instrument (MERSQI) scores were calculated [32]. As this was used to qualitatively assess the strength of the link between design characteristics and their effect, MERSQI scores were not calculated for the whole study, but only for the assessment of the effect on CRM/teamwork. The MERSQI [32] is considered a useful and reliable tool for appraising the methodological quality of medical education research with good interrater reliability [33]. The MERSQI has 6 domains (10 items) from study design to study outcome. Each domain has a maximum of 3 points that can be scored, and totals range from 5 up to 18 points. The 6 domains allow interpretation to focus on normative domain-specific scores, rather than on overall scores [33] and to better identify specific gaps in methodology. To maximize the available data, all studies were included in the analysis of effect and no cut-off scores were used to exclude studies based on their MERSQI score. The MERSQI scores were calculated by the first author (GJ). Additionally, three authors (JL, SB and WK) independently calculated a MERSQI score for 1 of the studies to check scoring quality and consistency. In case of doubt on the scoring in the other studies, this was solved by discussion in the full team of authors.

An Excel (Microsoft^®^ Excel^®^ for Microsoft 365 MSO, Redmond, Washington, U.S) data charting form was developed for data extraction and calculating MERSQI scores. The form was evaluated for consistency and completeness by extracting data from 3 included articles by 1 author (GJ), with double-checking by all other authors. After final amendments, 1 author (GJ) extracted the data.

### Synthesis of results

The three previously mentioned categories (study characteristics - educational and escape room design characteristics – effect on learning) were used as headings to summarize data. Data from studies in the virtual, student and setting group were included in the analyses and synthesis of the data was used to answer the research questions.

## Results

### Selection of sources of evidence

**Fig. 1:**
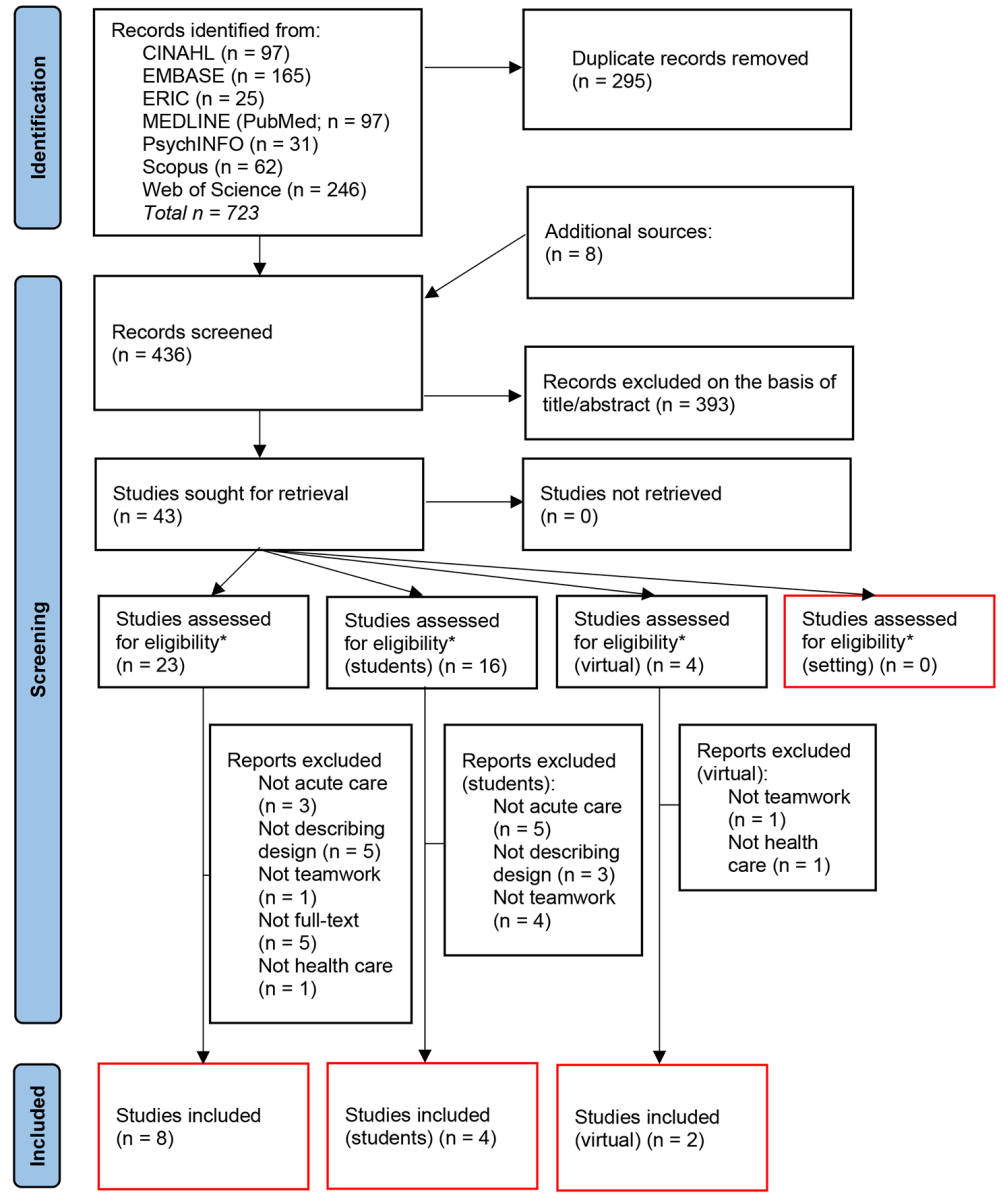
PRISMA flowchart of screening process [19]. *before the full text screening studies were divided into the different strands. If during full-text screening a study better fitted into one of the other strands, the study was transferred there

In Fig. 1a PRISMA flowchart [19] depicts the search results and screening process. Fourteen studies were included in the analysis, of which four were included in the ‘student’ group and two in the ‘virtual’, group. No studies were found for the ‘settings’ group. Of the fourteen studies, twelve described the design of an escape room. While the other two studies (both in the ‘student’ group) were evaluation studies of one of the escape rooms in the ‘student’ group.

### Qualitative Synthesis

The full data extraction form, including all extracted data can be found in the Additional file 2. The overall characteristics of the included studies are presented in Table 1. The two studies that only evaluated the effect of the escape room are displayed in relation with the study describing the escape room design (ID 10 A-B [34, 35]). In the 11 studies that evaluated the escape room, 419 participants were included (mean 45). In 50% of the studies (*n* = 6/12) the escape room was aimed at teamwork in Emergency Medicine. Aims of both the studies and the escape rooms differed significantly. Excluding both evaluation studies, two-third of the studies (*n* = 8/12) primarily described design [36–43], while the others [44–47] had evaluation as their primary aim. Teamwork was not the sole aim of all escape rooms, as eight studies also aimed at knowledge and/or skills. This heterogeneity complicated the extraction of design criteria, their underpinning and relating them to their effect on CRM/teamwork.

**Table 1:** Characteristics and description of included studies

ID	Author + year	Subjects (n)	Country	Setting	Study aim	ER aim	Aim^1^	Description	Evalua-tion^2^
1	Abensur Vuillaume [36]	N/A	France	EM	Describe steps in creating and implementing an ER	Team communication, knowledge and diagnostic process on non-traumatic coma, pulmonary embolism and nitric oxide poisoning	B	Descriptive study on a predefined 6 step method to develop an ER with 8 game play interactions and a 30-minute time limit	No
2	Borck [37]	57	USA	OR	Describe how an ER can support interprofessional and intergenerational learning	Improving interprofessional teamwork and multiple BLS/ALS skills	B	Descriptive study on development and evaluation of 3 sequential ERs, aimed at a CPR situation in the OR.	Yes S
3	Daniel [47]	66	USA	V	Combining an ER and simulation-based education to dynamically teach cardiac arrest management	Teamwork: information processing, identify problem solving strengths, communication, time-management. Outcomes: decision-making, dividing workload, shared mental model. Resuscitation skills	B	Cohort study describing effect on knowledge, skills and reaction of a mock code simulation ER. Nine puzzles and a 60-minute time limit	Yes S
4	Gomez-Urquiza [46]	50	Spain	ICU EM	Analyze the opinion of EM and ICU professionals on the use of ERs as an evaluation game	Not mentioned	B	Quantitative, descriptive, cross-sectional study to analyze the opinion of EM and ICU professionals on the use of ERs as an evaluation game	Yes S
5	Kutzin [38]	10	USA	V	Describe the planning, implementation, and evaluation of an ER scenario	Importance of teamwork and communication. Describe methods of team formation and leader’s emersion. Identify communication barriers in teamwork	T	Pilot study on the effect of an ER, consisting of 2 puzzle paths (3 and 4 puzzles), requiring minimal clinical knowledge. Time limit was 45 minutes	Yes S
6	Podlog [44]	30	USA	EM	An ER on EM content and procedural aptitude engages learners more than traditional didactics	Creating an engaging teambuilding ER to teach EM content and procedural aptitude	B	Short descriptive educational article on the use of an ER to teach EM knowledge and skills. The ER consisted of 8 ‘EM-content’ puzzles and had a 60-minute time limit	Yes S
7	Rosenkrantz [39]	29	Den-mark	EMS MS	Detailed description of an ER to create learning opportunities and evaluation in two settings	Apply non-technical skills (NTS) in a complex team-based situation	T	1. Description of ER development, consisting of 7 puzzles and a 45-minute time limit. 2. Qualitative triangulated observational study in EMS and MS	Yes S V
8	Sanders [40]	17	USA	EM	Detail the steps to set up a well working healthcare themed ER	To promote teamwork and communication	T	Descriptive study on the development of a medically themed ER, consisting of 8 puzzles and with a 20-minute time limit	Yes I
**Students**									
9	Holland [45]	85	USA	OB	Evaluate a healthcare simulation ER in maternity nursing students	Communication and collaboration in postpartum hemorrhage (PPH) scenarios	B	Mixed methods convergent parallel design study on the effect of a simulation ER. Theme: a PPH case, consisting of 7 puzzles	Yes S F
10	Morrell [41]	N/A	USA	NS	Detail a cardiovascular ER, incl. educational objectives, design considerations, and materials for transferability	Engage students in learning, identify areas of weakness, and build collaborative interaction	B	Descriptive study on the design and implementation of a cardiovascular themed ER, consisting of 9 puzzles and a 60-minute time limit	No
10A	Morrell – soft [35]	8	USA		Explore perceptions of the ER	As above		Basic interpretive qualitative methodology study using inductive and deductive content analysis	Yes F
10B	Morrell – escape [34]	4	USA		Evaluate impact of ER on knowledge and perceptions	As above		Non-experimental mixed-methods pilot study using concurrent triangulation. Pre-post knowledge tests and qualitative analysis of focus group discussion	Yes F
**Virtual**									
11	Kutzin [42]	U	USA	EM	Describe creation of a virtual ER and assessing usability and participant feelings	Introduce important teamwork and communication concepts	T	Descriptive study on the design of a virtual ‘zombie apocalypse’ ER, consisting of 5 puzzles and with a 30-minute time limit.	Yes S FF
12	Turner [43]	63	USA	EM FM	Describe background, set up and evaluation of a virtual emergency burn care ER	Teamwork: Communication and collaboration, task-switching and leadership skills. Multiple knowledge items: inhalation, intoxication and emergency burn care.	B	Descriptive study, providing background and practical guide on a virtual emergency burn care ER, consisting of 8 puzzles and a 15-minute time limit	Yes S

^1^aim of escape room: B = both teamwork and skills/knowledge, T = teamwork. ^2^Evaluation: yes/no + method: F = focus group, FF = formative feedback, I = Informal group evaluation, S = survey, V = video analysis. Other abbreviations: ALS = Advanced Life Support; BLS = Basic Life Support; EM = Emergency Medicine; EMS = Emergency Medical Services; ER = Escape room; FM = Family Medicine; ICU = Intensive Care Unit; MS = Medical Students; NS = Nursing Students; NTS = non-technical skills; OB = Obstetrics; OR = Operating Room; PPH = postpartum hemorrhage; U = unknown; V = Varied

### Educational and escape room design characteristics

The underlying pedagogical or didactical principles and/or learning theories were only noted in four of the twelve studies. Specifically, Kutzin [38] provided an elaborate underpinning using interdisciplinary game theory for the development of his physical escape room aimed at teamwork. Rosenkrantz [39] Sanders [40] and Kutzin (virtual) [42] gave a short explanation for using the concept of edutainment in the development of their escape rooms. Four studies provided a theoretical CRM/teamwork framework with which the escape room was developed. Kutzin [38], Sanders [40] and Kutzin (virtual) [42] used the TeamSTEPPS framework and Rosenkrantz used the ‘Anesthesiologists Non-Technical Skills in Denmark (ANTSdk)’. Of the other eight studies, Turner [43] and Daniel [47] mentioned a number of non-technical skills like task-switching, leadership and shared mental model as a learning goal. The escape room learning goal of improving communication and/or teamwork was not further specified in all other studies.

Table 2 shows the summarized data that was extracted on escape room design. Full data on all studies can be found in Additional file 2. Teams were given median 38 min (range 15–60) to escape. Teams ranged from 5 to 10 participants, with 4–6 being the most common team size (50%; *n* = 6/12); in three (25%) studies team size was not mentioned [36, 37, 40].

**Table 2:** Escape room design

ID	Puzzle Organisation^1^	Alignment with teamwork learning goals	Design + testing	Briefing	Debriefing structure	Guidance by facilitator/moderator	Team size	Time to escape (min)	Theme
1	L	Combine the educational objectives of gameplay with its interactive elements	Multidisciplinary team using 6-step method. Game test by 2 teams	Video	By facilitator. Guideline: group’s opinion on communication, incl. strengths and weaknesses. Relate to clinical situation	Present in room. Intervene at points and needed for additional information	-	30	Medical
2	-	-	Workgroup identified learning outcomes for escape room. Manual written	Scenario given	-	Support the learners to escape in time	-	-	Medical
3	L	-	Piloted with novice nurses	10-min in classroom	PEARLS tool and the Plus/ Delta/ Gamma framework	Silent in corner. Can be asked for additional clue	Av. 8	60	Medical
4	C	-	-	-	-	Provide 1 clue after 10 min., and 1 after 20 min.	5	30	Medical
5	P	-	-	Introduction to concept of ER	By facilitator: team dynamics/ cohesion/ forming, leadership, and communication. Mentioning TeamSTEPPS moments; relate to clinical situation	-	10	45	Medical and non-medical
6	P	-	Piloted to check sufficient escape time	Briefing on the activity outside the ER	Answers and a brief explanation to puzzles	Available for subtle clues if stuck	10	60	Medical
7	L	-	Extensive manual incl. hints and briefing instructions. Iterative testing with refinement	Short explanation by facilitator and video	Not done	Hints provided in manual, including timing to give them	5	45	Medical
8	O L	Multiple puzzles could be done at once, so teams needed to communicate	-	Facilitator: introduce scenario, explain rules, maintain safe environment	1. Walk-through of the ER. 2. Debriefing using TeamSTEPPS® and medical content. 3. Discuss partially solved clues and improving communication. 4. relate to clinical situation	Facilitator in the room providing clues as needed or on request	-	20	Medical
**Students**									
9	L	Following Healthcare Simulation Standards of Best Practice in Simulation Design	By design team. Testing: Instructor observations and observational and formal student feedback	Instructions, expectations, possibility to leave and safe learning environment	Debriefing with Good Judgment Model. Semi-structured interview guide promoting reflective thinking and guided reflection. Also used as focus group	Providing clues on request. One free clue, following clue added a minute to ER time	4–5	-	Medical
10	L	-	Implemented, with game changes after feedback. Then pilot tested.	Video trailer and reading of scenario and game rules	Guided debriefing on content and soft skills. Questions on game tasks, teamwork behaviors, communication, experiences of providing feedback, and perceptions about help-seeking	In the room for progress tracking and assist as needed. Groups get 4 helps: nurse assistance, physician input, pharmacy, or medical library	4–5	60	Medical
**Virtual**									
11	C	Phone/computer gathered information, required additional communication in team	-. However, all facilitators had to participate in the room	Overview video providing backstory	Experienced simulation educator discussed uncovered TeamSTEPPS principles and related them to clinical situation	Trained by completing ER. Provide link to 1 participant. Give hints as needed. Backed up by full guidebook	4–6	30	Non-medical
12	L	Communication: use team knowledge. Resourcefulness: arrive at solutions. Task-switching: look up information and communicate with team. Leadership skills for effective teamwork. Varying question format: mimic physical ER, encourage brainstorming, thinking aloud, and teamwork.	-	PowerPoint introduction (10min)	5 minutes. Proposed questions “How was the experience? What went well? What did not?”	Separate attendees into groups. Rotate through groups for technical questions, ensure rules are followed, observe team dynamics for feedback	4–6	15	Medical

^1^Puzzle organization: C = Complex; L = linear; O = Open, P = path-based; ‘-‘ = not mentioned. Abbreviations: Av. = Average; CPR = Cardio-Pulmonary Resuscitation; ECG = Electrocardiogram; ER = Escape Room; Incl. = Including; PEARLS = Promoting Excellence and Reflective Learning in Simulation; ‘-‘ = not mentioned

It is noteworthy that data on the alignment of the escape room with the teamwork learning objectives could only be extracted from five studies, with only Turner [43] providing specifics on each teamwork item. Alignment with skills and knowledge training was either explicitly stated and/or could be inferred from the puzzle theme or description (data not included). However, how the need for teamwork was ensured or facilitated was often not mentioned, other than that escape rooms were collaborative by nature.

With regard to the organization of the puzzles, the linear path (also called sequential) was most common (*n* = 7/12), with 1 study combining the open and linear path [40].

Seven of the twelve studies noted a structured debriefing with the use of guidelines and known debriefing tools. Two studies did not mention whether debriefing was used and one study specifically mentions there was no debriefing. The two remaining studies mentioned a short debrief mainly aimed at the answers to the puzzles.

For interprofessional education we extracted data on the interdependence and embodiment (immersion). Interdependence was not specifically mentioned in the included studies. However, in four studies data were found that indicated some degree of interdependence. Sanders [40] mentioned presenting multiple puzzles at once, so different team members could be engaged and work simultaneously on different puzzles. Additionally, the team was led to certain points where they had to work together as a full team. The virtual escape room by Kutzin (virtual) [42] had several puzzles that ‘required participants to work on different screens with a need to communicate’. Additionally in the escape rooms developed by Podlog [44] and Gomez-Urquiza [46], the puzzle scheme allowed for participants to work on several puzzles simultaneously.

Details on immersion could be extracted from five studies and was often accomplished by using attributes and props that were also found in daily practice, and/or that were related to the escape room theme. Additionally darkening of the room was used by Rosenkrantz [39] and Sanders [40]. Sanders [40] and Abensur Vuillaume [36] mentioned using their escape room introduction to set the theme.

Escape rooms can be used as stand-alone activities, but are also often integrated into a course, curriculum, or used in combination with other teaching modalities. Three studies used the escape room as a stand-alone activity [37, 39, 46], two studies did not mention other teaching modalities [36, 42], while the others used one or more other teaching modalities. Sanders, for example, used the escape room as part of an annual competition among pediatric Emergency Medicine faculty and fellows [40], while Kutzin [38] and Holland [45] used the escape room as part of an obligatory course. Morrell used a broad range of other teaching modalities: lectures, activities, case studies, videos, assigned readings, and simulation [41]. The escape room by Daniel had ALS simulations before and after the escape room [47]. Both Podlog [44] and Turner [43] tried to increase knowledge retention by giving a didactic summary and lecture respectively.

### Effect on learning

Effectiveness was investigated in 13 studies. The data from Rosenkrantz [39] was split into acute care personnel and students as different methods were used to study the effect of their escape room in these 2 groups. See Table 1 for the measures used per study. Most studies (*n* = 10) studied reaction (level 1) to the escape room using surveys (*n* = 9), or informal feedback (*n* = 1). Specific phrasing and number of questions differed, with 3 out of 9 survey studies not providing the specific questions [37, 42, 44]. In general participants were asked whether they enjoyed the escape room and thought it was effective in training teamwork.

Holland [45], besides using a survey, and both studies by Morrell [34, 35] used focus group analysis to study modification of attitudes/perceptions (level 2a). Rosenkrantz [39], besides using a survey, rated videos of the fastest and slowest student group on the use of non-technical skills, which constituted studying acquisition of knowledge/skills (level 2b).

All the included studies showed a positive effect on teamwork of the escape room on the levels of effect they scored on. In the ten studies scoring Kirkpatrick level 1 > 80% of participants scored positive on enjoyment and engagement. Participants also generally felt that teamwork was trained by the escape room with mean Likert scores > 80% of the maximum, and teamwork and communication were often mentioned in response to open-ended questions directed at what participants felt they had learned. In the analysis on learning outcomes from focus groups (Kirkpatrick level 2a), teamwork also emerged as a theme in all 3 studies. Rosenkrantz [39] noted in the assessment of videos of students (Kirkpatrick level 2b) that time to finish the escape room was not related to whether there was a team leader. Video observers in the same study rated gathering information, exchanging information and reassessing decisions as the most used non-technical skills.

Additional file 3 shows the MERSQI scores of all the included studies. The median MERSQI score is 7.0 (range 6.0–12.0), which is lower than the mean of 9.6 in the original paper where MERSQI was first described [32]. Notably ‘validity evidence for evaluation instrument scores’ were rather low, with only 5 studies scoring 1.0 out of 3.0 points. This relates to the fact that researchers mostly used surveys that were developed for their study.

## Discussion

In this scoping review of fourteen studies addressing the design and effect of escape rooms used for training CRM/teamwork for healthcare professionals in acute care, we identified several commonly used design requirements, but noticed a lack of reporting on alignment and insufficient data to connect design requirements to learning outcomes. Below, these results are discussed per research question.

### Design characteristics

Common design characteristics were derived from the whole group of included studies, given the small number of studies. Team size (4–6 participants) was in line with commonly used team sizes in healthcare and other fields [12, 17]. The time limit of about 40 min was slightly shorter compared to the 60 min found in earlier reviews on escape rooms [12, 17]. We found Puzzle organization most often to be linear, though complex and open puzzle organization were explicitly used to evoke teamwork. Some form of briefing and use of a facilitators (inside or outside the escape room) to moderate progress and provide hints were also used in all studies. Debriefing was often structured, coupling teamwork factors to what happened during the escape room and often relating this to the clinical situation. This suggests that debriefing was used to critically reflect on CRM/teamwork and promote learning. This parallels to simulation, where debriefing is considered a key factor in learning [48].

Little information was given on the alignment of the escape room with the teamwork learning goals. This may be due to the study types, which commonly focused on the effect of the escape room, instead of on how design moderated or optimized teamwork learning outcomes. However, even in those studies focused on design [36, 39–41], little was reported on how design characteristics were used to achieve the desired teamwork learning outcomes. This was also reflected in the other data-items which were often not reported or could only be extracted indirectly.

### Design characteristics and learning outcomes: deriving design requirements

In an attempt to connect design characteristics to their effect on learning outcomes, we reviewed the outcome measures and effect of the included escape rooms. These outcomes reflected the potential escape rooms yield for teamwork training, as reactions (level 1) to the escape room were overtly positive in all studies. This was further strengthened by the effects, be it in small numbers, seen on modification of attitudes/perceptions (level 2a) and studying acquisition of knowledge/skills (level 2b). However, looking at the quality of this evidence, there is no evidence on higher Kirkpatrick levels and study sizes are rather small. Additionally MERSQI scores on determining CRM/teamwork learning outcomes were rather low (median 7.0; mean 7.8), compared to for example the 9.6 mean in the original paper where MERSQI was first described [32]. Within the MERSQI the low ‘validity evidence for evaluation instrument scores’ further underscores the lack of valid effect measures. Combined with the considerable study heterogeneity, this precluded any conclusions on the effect design characteristics had on learning outcomes and the deriving of design requirements.

### Gaps and looking forward

From our results we see 2 main gaps in the design of escape rooms aimed at CRM/teamwork in acute care professionals: (i) a lack of data on the effect of design requirements on learning outcome and (ii) a lack of (reporting on) alignment between learning goals and design of these escape rooms.

i) Escape rooms are collaborative by nature, with teams communicating and working on puzzles together. This suggests teamwork and attests to the potential escape rooms have. The overtly positive effects that were seen in the included studies confirmed this, and is in line with data from reviews covering a broad range of educational fields [17, 24], including healthcare students [12]. In acute care this positive effect was also seen in two studies on the effect of a commercial escape room on teamwork [49, 50]. Especially Valdes et al. [49], who showed that key CRM aspects improved in acute care simulation after participation in an escape room, attest to the potential escape rooms have for CRM/teamwork in acute care. However, which design characteristics maximize this potential remains unknown. None of the studies described or examined which characteristics were key in reaching the desired learning outcomes. As an example, we found the linear puzzle organization (in which one puzzle leads to the other) to be the most common. In their systematic review Veldkamp et al. [17], however stated that team-based medical escape rooms do not align well with a linear puzzle organization and suggested using other organizations. Whether different puzzle organizations really lead to higher learning outcomes, is debatable as they have not been compared directly. However, we do agree with their conclusion that studying the effect of different design characteristics on learning outcomes is necessary to maximize learning outcomes. A first step is systematic reporting on these design characteristics in all studies using escape rooms to train CRM/teamwork learning in healthcare. Reporting the data items we extracted, which are in line with a range of escape room development frameworks [14–16], would be a good way to start. Future research should not only focus on the effect escape rooms have, but also on the mechanisms by which this effect is reached, comparing different design characteristics and extracting which are pivotal and therefore should be considered as design requirements. By identifying these requirements, an evidence-based foundation can be laid for developing and executing these escape rooms.
ii) The need for effective design requirements relates to the other major gap we identified: the need for better (reporting on) alignment between learning goals and escape room design. As is also acknowledged by others [16, 51], we agree that better alignment should be sought, investigated and reported [17]. Cohen et al. [16] provide design considerations for escape rooms aimed at teamwork and advice using the Input-Moderator-Output-Input model by Ilgen [52] to identify and measure a variety of factors that best predict team outcomes and others advocate the use of Educational Design Research (EDR) [53]. Both could be used to initiate iterative cycles of individual puzzle or complete escape room development, leading not only to better reporting of alignment, but also to more effective design and the development of design requirements.

### Strengths and limitations

Educational escape rooms are a relatively young and growing field. The fact that this field is young, translated into a limitation for our review. Only a relatively small number of studies with modest population size and limited methodological quality fitted our criteria. We therefore included studies on virtual acute care escape rooms and acute care escape rooms in students, which allowed us to enrich this dataset. By clearly marking these groups, it is easily deducible where the data came from.

Less than half of the studies had description of design as their primary aim, limiting the data that could be extracted. However, looking from an educational perspective, this review offers an excellent new starting point in the iterative cycle of development. A final limitation is that the search was conducted in June 2023 and research published since is not included.

Despite the above limitations, to our knowledge, the present study is the first to provide a comprehensive analysis of escape room design aimed at enhancing CRM/teamwork in acute care professionals.

## Conclusion

In conclusion we found that escape rooms that aim at improving CRM/teamwork in acute care professionals often have 4–6 participants, a 40-minute time limit, linear puzzle organization, use briefing and a structured debriefing is considered important for learning. Reporting on alignment of CRM/teamwork learning goals and escape room design is insufficient and there is insufficient evidence on how and whether design characteristics optimize learning outcomes. There is a need for more complete reporting of future escape rooms aimed at training teamwork in acute care professionals and research on maximizing the learning potential of these escape rooms through design.

### Electronic supplementary material

Below is the link to the electronic supplementary material.

Supplementary Material 1: Selection of databases and search strategy. Additional information on the selection of databases, the used additional resources and the full search strings for all databases.

Supplementary Material 2: Data extraction form. The full data extraction form (Table 1), including all data and data-items that were extracted (data-items are headings of the different columns). Calculated MERSQI score (Table 2).

Supplementary Material 3: Table with MERSQI scores. A Table with the full MERSQI scores, including scores on all separate items.

## Data Availability

All data generated or analyzed during this study are included in this published article and its supplementary information files (additional file 2).

## Abbreviations

ALS: Advanced Life Support
Av: Average
BLS: Basic Life Support
CPR: Cardio-Pulmonary Resuscitation
CRM: Crew Resource Management
ECG: Electrocardiogram
EDR: Educational Design Research
EM: Emergency Medicine
EMS: EMS Emergency Medical Services
ER: Escape Room
FM: Family Medicine
Incl: Including
ICU: Intensive Care Unit
MERSQI: Medical Education Research Study Quality Instrument
MS: Medical Students.
NS: Nursing Students
NTS: Non-technical skills
OB: Obstetrics
OR: Operating Room
PEARLS: Promoting Excellence and Reflective Learning in Simulation
PPH: Postpartum Hemorrhage
U: Unknown
V: Varied
