# Supplementary material for: Escape room design in training crew resource management in acute care: a scoping review
Source: BMC Med Educ. 2024 Jul 30;24:819. doi: 10.1186/s12909-024-05753-z (PMC11290095; doi:10.1186/s12909-024-05753-z)
Supplement: Supplementary file 1 — Supplementary Material 1: Selection of databases and search strategy. Additional information on the selection of databases, the used additional resources and the full search strings for all databases. [file 12909_2024_5753_MOESM1_ESM.docx]

## Additional file 1. Selection of databases and search strategy

Training CRM with escape rooms in healthcare has aspects of psychology, education and medicine. Databases were therefore selected to cover these subjects. Additionally, Web of science, a large database covering all scientific areas, was also selected to include other areas and not miss any relevant literature. Next to the databases additional resources were also searched (see ‘additional resources’ below). The following databases were selected:

- CINAHL
- EMBASE
- ERIC
- MEDLINE (PubMed)
- PsycINFO
- Scopus
- Web of Science

Additional resources:

To cross-check that no relevant articles were missed, we used several other methods to check the search results.

- Elicit.org. Elicit is an AI tool that uses language model to, amongst other things, find relevant papers. As it uses algorithms based on questions asked in natural language, it cannot be used for systematic searches. One author (GJ) posed different questions and checked the results against the systematic search result.
- Google Scholar. As Google Scholar does not support full Boolean logic or wild cards and uses ranking algorithm, it is difficult to use for systematic searches. However, as it is, compared to the other databases, less limited in the sources it searches, it is possible that articles, that were missed in the systematic search, are found. One author (GJ) therefore used several combinations of keywords and checked the results against the search results and eligibility criteria.
- Of articles eligible for inclusion:
  - Forward citation search in Web of Science was used to search for additional eligible articles.
  - Reference lists were checked for articles eligible for inclusion (backward citation search)

Date of most recent search: 28-06-2023

The search was drafted by GJ and refined through team discussion. A librarian, experienced in systematic searches, evaluated and further refined the search strategy.

### Search strings for the separate databases

#### Pubmed

("escape room*"[tiab] OR "escape game*"[tiab] OR "escape box*"[tiab] OR "puzzle room*"[tiab] OR "escape activit*"[tiab])

AND

(CRM[tiab] OR "Crew resource*"[tiab] OR "cockpit resource*" [tiab] OR "crisis resource*"[tiab] OR team*[tiab] OR "human factor*"[tiab] OR "non-technical skill*"[tiab] OR Crew Resource Management, Healthcare[mesh] OR situation* awareness*[tiab] OR task allocation*[tiab] OR decision making[tiab] OR communication[tiab] OR leadership[tiab] OR leadership[mesh])

AND

Limit to year >2000

#### Embase

("escape room*".ti,ab,kf. OR "escape game*" .ti,ab,kf. OR "escape box*".ti,ab,kf. OR "puzzle room*".ti,ab,kf. OR "escape activit*".ti,ab,kf. OR "break out room*".ti,ab,kf.)

AND

(health care personnel management/ OR teamwork/ OR CRM.ti,ab,kf. OR "Crew resource*".ti,ab,kf. OR cockpit resource*.ti,ab,kf. OR "crisis resource*".ti,ab,kf. OR team*.ti,ab,kf. OR "human factor*".ti,ab,kf. OR "non-technical skill*".ti,ab,kf. OR situation* awareness*.ti,ab,kf. OR task allocation*.ti,ab,kf. OR decision making.ti,ab,kf. OR communication.ti,ab,kf. OR leadership/ OR leadership.ti,ab,kf.)

AND

Limit to year >2000

#### Web of Science

(TS = ("escape room*" OR "escape game*" OR "escape box*" OR "puzzle room*" OR "escape activit*" OR "mystery room*" OR "break out box*" OR "break out room*"))

AND

(TS = (CRM OR "Crew resource*" OR "cockpit resource*" OR "crisis resource*" OR team* OR "human factor*" OR "non-technical skill*" OR "situation* awareness*" OR teamwork* OR "task allocation*" OR "decision making" OR communication OR leadership))

AND

Limit to year >2000

#### Scopus

(TITLE-ABS-KEY("escape room*") OR TITLE-ABS-KEY("escape game*") OR TITLE-ABS-KEY("escape box*") OR TITLE-ABS-KEY("puzzle room*") OR TITLE-ABS-KEY("escape activit*") OR TITLE-ABS-KEY("mystery room*") OR TITLE-ABS-KEY("break out box*") OR TITLE-ABS-KEY("break out room*"))
AND
(TITLE-ABS-KEY(CRM) OR TITLE-ABS-KEY("Crew resource*") OR TITLE-ABS-KEY("cockpit resource*") OR TITLE-ABS-KEY("crisis resource*") OR TITLE-ABS-KEY(team*) OR TITLE-ABS-KEY("human factor*") OR TITLE-ABS-KEY("non-technical skill*") OR TITLE-ABS-KEY("situation* awareness*") OR TITLE-ABS-KEY("task allocation*") OR TITLE-ABS-KEY("decision making") OR TITLE-ABS-KEY(communication) OR TITLE-ABS-KEY(leadership))

AND

Limit to year >2000

#### CINAHL

((TI "escape room*" OR AB "escape room*" OR SU "escape room*") OR (TI "escape game*" OR AB "escape game*" OR SU "escape game*") OR (TI "escape box*" OR AB "escape box*") OR (TI "puzzle room*" OR AB "puzzle room*" OR SU "puzzle room*") OR (TI "escape activit*" OR AB "escape activit*" OR SU "escape activit*") OR (TI "break out room*" OR AB "break out room*" OR SU "break out room*"))
AND
((MH teamwork) OR (TI CRM OR AB CRM OR SU CRM) OR (TI "Crew resource*" OR AB "Crew resource*" OR SU "Crew resource*") OR (TI "cockpit resource*" OR AB "cockpit resource*" OR SU "cockpit resource*") OR (TI "crisis resource*" OR AB "crisis resource*" OR SU "crisis resource*") OR (TI team* OR AB team* OR SU team*) OR (TI "human factor*" OR AB "human factor*" OR SU "human factor*") OR (TI "non-technical skill*" OR AB "non-technical skill*" OR SU "non-technical skill*") OR (TI "situation* awareness*" OR AB "situation* awareness*" OR SU "situation* awareness*") OR (TI "task allocation*" OR AB "task allocation*" OR SU "task allocation*") OR (TI "decision making" OR AB "decision making" OR SU "decision making") OR (TI communication OR AB communication OR SU communication) OR (TI leadership OR AB leadership OR SU leadership) OR (MH leadership))

AND

Limit to year >2000

#### ERIC

("escape room*".ti,ab,id. OR "escape game*".ti,ab,id. OR "escape box*".ti,ab,id. OR "puzzle room*".ti,ab,id. OR "escape activit*".ti,ab,id. OR "mystery room*".ti,ab,id. OR "break out room*".ti,ab,id. OR "break out box*".ti,ab,id.)
AND
(teamwork/ OR Soft Skills/ OR Team Training/ OR leadership/ OR decision making/ OR human factors engineering/ OR CRM.ti,ab,id. OR "Crew resource*".ti,ab,id. OR "cockpit resource*".ti,ab,id. OR "crisis resource*".ti,ab,id. OR team*.ti,ab,id. OR "human factor*".ti,ab,id. OR "non-technical skill*".ti,ab,id. OR "situation* awareness*".ti,ab,id. OR "task allocation*".ti,ab,id. OR "decision making".ti,ab,id. OR communication.ti,ab,id. OR leadership.ti,ab,id.)

AND

Limit to year >2000

#### Psychinfo

("escape room*".ti,ab,id. OR "escape game*".ti,ab,id. OR "escape box*".ti,ab,id. OR "puzzle room*".ti,ab,id. OR "escape activit*".ti,ab,id. OR "break out room*".ti,ab,id. OR "break out box*".ti,ab,id.)
AND
(exp Human Factors Engineering/ OR exp teamwork/ OR Interpersonal Interaction/ OR Collaboration/ OR exp leadership/ OR CRM.ti,ab,id. OR Group Dynamics/ OR Group Performance/ OR Interdependence/ OR exp Interpersonal Communication/ OR "Crew resource*".ti,ab,id. OR "cockpit resource*".ti,ab,id. OR "crisis resource*".ti,ab,id. OR team*.ti,ab,id. OR "human factor*".ti,ab,id. OR "non-technical skill*".ti,ab,id. OR "situation* awareness*".ti,ab,id. OR "task allocation*".ti,ab,id. OR "decision making".ti,ab,id. OR communication.ti,ab,id. OR leadership.ti,ab,id.)

AND

Limit to year >2000
