# Supplementary material for: Escape room design in training crew resource management in acute care: a scoping review
Source: BMC Med Educ. 2024 Jul 30;24:819. doi: 10.1186/s12909-024-05753-z (PMC11290095; doi:10.1186/s12909-024-05753-z)
Supplement: Supplementary file 3 — Supplementary Material 3: Table with MERSQI scores. A Table with the full MERSQI scores, including scores on all separate items. [file 12909_2024_5753_MOESM3_ESM.docx]

# Additional file 3: table with MERSQI scores

| Domain | | Study design | Sampling | | Type of data | Validity evidence for evaluation instrument scores | | | Data analysis | | Out-come |  |
| --- | --- | --- | --- | --- | --- | --- | --- | --- | --- | --- | --- | --- |
| Item | ID |  | Institu-tions | Res-ponse rate |  | Content | Internal struc-ture | Relationship to other variables | Appro-priate | Complexity/ sophistica-tion |  | **Total score** |
|  | 1 |  |  |  |  |  |  |  |  |  |  | N/A |
|  | 2 | 1.0 | 0.5 | 1.5 | 1.0 | 0.0 | 0.0 | 0.0 | 0.0 | 1.0 | 1.0 | 6.0 |
|  | 3 | 1.0 | 0.5 | 1.5 | 1.0 | 1.0 | 0.0 | 0.0 | 1.0 | 1.0 | 1.0 | 8.0 |
|  | 4 | 1.0 | 0.5 | 1.5 | 1.0 | 0.0 | 0.0 | 0.0 | 1.0 | 1.0 | 1.0 | 7.0 |
|  | 5 | 1.0 | 0.5 | 1.5 | 1.0 | 0.0 | 0.0 | 0.0 | 1.0 | 1.0 | 1.0 | 7.0 |
|  | 6 | 1.0 | 0.5 | 1.5 | 1.0 | 0.0 | 0.0 | 0.0 | 1.0 | 1.0 | 1.0 | 7.0 |
|  | 7 | 1.0 | 0.5 | 1.0 | 1.0 | 0.0 | 0.0 | 0.0 | 1.0 | 1.0 | 1.0 | 6.5 |
|  | 8 | 1.0 | 0.5 | 1.5 | 1.0 | 0.0 | 0.0 | 0.0 | 0.0 | 1.0 | 1.0 | 6.0 |
| Students | |  |  |  |  |  |  |  |  |  |  |  |
|  | 9 | 1.0 | 1.0 | 1.5 | 3.0 | 1.0 | 0.0 | 0.0 | 1.0 | 2.0 | 1.5 | 12.0 |
|  | 10 |  |  |  |  |  |  |  |  |  |  | N/A |
|  | 10A | 1.0 | 0.5 | 0.5 | 3.0 | 1.0 | 0.0 | 0.0 | 1.0 | 1.0 | 1.5 | 9.5 |
|  | 10B | 1.0 | 0.5 | 0.5 | 3.0 | 1.0 | 0.0 | 0.0 | 1.0 | 1.0 | 1.5 | 9.5 |
|  | 7 | 1.0 | 0.5 | 1.5 | 3.0 | 0.0 | 0.0 | 0.0 | 1.0 | 1.0 | 1.5 | 9.5 |
| Virtual | |  |  |  |  |  |  |  |  |  |  |  |
|  | 11 | 1.0 | 0.5 | N/A | 1.0 | 0.0 | 0.0 | 0.0 | 1.0 | 1.0 | 1.0 | 6.0 |
|  | 12 | 1.0 | 0.5 | 0.5 | 1.0 | 1.0 | 0.0 | 0.0 | 1.0 | 1.0 | 1.0 | 7.0 |

Table. MERSQI scores of effectiveness studies. Scores were calculated only for the measurement of the effect on CRM/teamwork. N/A = not applicable
